# Ultrasensitive Detection of COVID-19 Causative Virus (SARS-CoV-2) Spike Protein Using Laser Induced Graphene Field-Effect Transistor

**DOI:** 10.3390/molecules26226947

**Published:** 2021-11-17

**Authors:** Tian-Rui Cui, Yan-Cong Qiao, Jian-Wei Gao, Chun-Hua Wang, Yu Zhang, Lin Han, Yi Yang, Tian-Ling Ren

**Affiliations:** 1 School of Integrated Circuit, Tsinghua University, Beijing 100084, China; ctr19@mails.tsinghua.edu.cn (T.-R.C.); qyc16@mails.tsinghua.edu.cn (Y.-C.Q.); 2 Beijing National Research Center for Information Science and Technology (BNRist), Tsinghua University, Beijing 100084, China; 3 Institute of Marine Science and Technology, Shandong University, Qingdao 266237, China; gaoxiii@foxmail.com (J.-W.G.); wangchunhua@mail.sdu.edu.cn (C.-H.W.); yuzhang@sdu.edu.cn (Y.Z.); 4 Center for Flexible Electronics Technology, Tsinghua University, Beijing 100084, China

**Keywords:** LIG-FET, biosensor, COVID-19, SARS-CoV-2, flexible devices

## Abstract

COVID-19 is a highly contagious human infectious disease caused by the severe acute respiratory syndrome coronavirus 2 (SARS-CoV-2), and the war with the virus is still underway. Since no specific drugs have been made available yet and there is an imbalance between supply and demand for vaccines, early diagnosis and isolation are essential to control the outbreak. Current nucleic acid testing methods require high sample quality and laboratory conditions, which cannot meet flexible applications. Here, we report a laser-induced graphene field-effect transistor (LIG-FET) for detecting SARS-CoV-2. The FET was manufactured by different reduction degree LIG, with an oyster reef-like porous graphene channel to enrich the binding point between the virus protein and sensing area. After immobilizing specific antibodies in the channel, the FET can detect the SARS-CoV-2 spike protein in 15 min at a concentration of 1 pg/mL in phosphate-buffered saline (PBS) and 1 ng/mL in human serum. In addition, the sensor shows great specificity to the spike protein of SARS-CoV-2. Our sensors can realize fast production for COVID-19 rapid testing, as each LIG-FET can be fabricated by a laser platform in seconds. It is the first time that LIG has realized a virus sensing FET without any sample pretreatment or labeling, which paves the way for low-cost and rapid detection of COVID-19.

## 1. Introduction

Coronavirus disease 2019 (COVID-19) is a newly emerging human infectious disease associated with severe respiratory distress. In December 2019, after a series of cases of pneumonia of unknown cause were reported [1], the world started a protracted struggle against the epidemic [2]. As human-to-human transmission rapidly increased, the World Health Organization (WHO) classified the COVID-19 outbreak as a pandemic [3]. Although the virus is under control to a certain extent regionally, there are still over 2.9 million new cases reported in just one week, with 49,000 new deaths [4], which brings the global cumulative number to 243 million since the beginning of the pandemic. Though many countries have rushed to prevent the spread of COVID-19, and some of which have mass vaccinations, no specific drugs are yet available, and there is an imbalance between supply and demand for vaccines. Under these circumstances, inspection and quarantine are the most effective way to control the epidemic [5,6,7]. Therefore, a cheap and fast detecting method is in urgent need.

**Figure 1:**
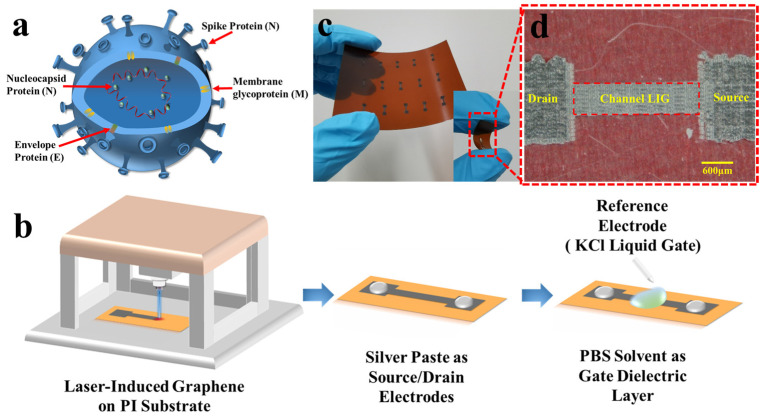
(**a**) Schematic illustration of SARS-CoV-2 with the composition of spike protein (S), membrane glycoprotein (M), nucleocapsid protein (N), and envelope protein (E). (**b**) Fabrication process of the LIG-FET. At the end of the process, the device was passivated with the SU-8 photoresist to decrease the leakage voltage and define the region of the liquid gate dielectric layer. (**c**) Optical image of the LIG-FET detector array showing high uniformity and flexibility. (**d**) Image of the LIG-FET with the channel region patterned by 840 mW laser, and the source/drain region by 900 mW.

The severe acute respiratory syndrome coronavirus 2 (SARS-CoV-2), as the RNA pathogenic virus of COVID-19, has a single-stranded RNA and four structural proteins: spike (S), envelope (E), matrix (M), and nucleocapsid (N) (Figure 1a) [8,9,10], which are the main detection targets of the virus. Currently, real-time reverse transcription-polymerase chain reaction (RT-PCR) is the golden standard of diagnosis [11,12]. However, molecular diagnosis using RT-PCR mostly takes at least 4–6 h, and its diagnostic accuracy is severely affected by the RNA preparation step [13]. Hence, highly sensitive diagnostic methods, which can directly detect viral antigens in clinical samples without pre-preparation steps, are necessary for rapid and accurate diagnosis of COVID-19. Based on the specific protein structure of SARS-CoV-2, protein detection is a good choice. The spike protein is the largest of the four structural proteins, which guides the virus to attach to the host cell. Its fundamental role in infectivity suggests that spike protein can be selected as a priority target for the detection of COVID-19 [14].

Up to now, different ways have been designed to analyze proteins. Based on the unique physical/chemical properties of the protein, there are techniques, such as electrophoresis [15], immunoblotting [16], autoradiography [17], mass spectrometry [18], and proteomics [19]. Among them, fluorescent tagging [20,21,22] or electrochemical detection [23,24,25] are the two main methods in the coronavirus-specific detection field. Fluorescence tagging, which depends on the composites in the reaction solution, has its fluorescence intensity affected by the distance of detecting particles, and the applications are hindered by the laboriousness and expensive reagent requirements [26]. Compared with fluorescent methods, the electrochemical detection has been widely developed since it requires minimal instrumentation, and it is scalable and can easily integrate with microelectronics. Moreover, some studies have focused on the label-free detection of biological molecules [27,28,29,30,31]. As a representative label-free detection tool in recent years, field-effect transistors (FETs), which utilize the electrical response to target molecules, can realize simple and fast detection of biomolecules [32,33,34]. In the FET-based biosensor, signals caused by the interactions between biomolecules on sensing interfaces are transformed into readable electrical signals and amplified with high sensitivity and good specificity [35]. FET-based biosensors have such advantages, including rapid detection speed, fast response time, and low detection limit. To fully realize those advantages, special attention needs to be paid to the channel materials.

Graphene is a two-dimensional sheet consisting of hexagonally arranged carbon atoms, which are all exposed on its surface, and biological molecules can be strongly absorbed to its surface through interactions, suitable to be used as the binding point of structure protein [36]. Furthermore, graphene is chemically inert, so specific receptors need to be modified on its surface to achieve highly selective recognition of targets. Among them, the non-covalent modification method can ensure that the electrical properties of graphene are not affected when the receptor is introduced, because it does not destroy the graphene lattice [37]. Moreover, with lower binding energy than the covalent bonding, the π-π stacking can satisfy most test environments [38], which means the graphene–bioreceptor interactions can be realized by the π-π stacking more easily. Meanwhile, the process of non-covalent bond modification is easy to control, and the modification results can be characterized accurately, which is beneficial to reduce the difficulty of the sensor design. Therefore, a graphene-based FET is an attractive choice for applications related to sensitive immunological diagnosis [39], and the method of obtaining graphene and the structure of the channel are the main influence factors.

Compared with other methods to produce graphene, such as mechanical exfoliation [40], chemical vapor deposition (CVD) [41], and reduction of graphene oxide (GO) [42], which are restricted by their relatively low-yield production, and cannot achieve preparation and patterning simultaneously, laser-inducing technology has been demonstrated as an efficient method to fabricate graphene-based devices [43]. In 2014, Lin et al. [44] creatively developed a method of converting polyimide (PI) into porous graphene via a CO_2_ infrared laser. This method is further applied to develop supercapacitors [45], flexible strain sensors [46], and an LIG artificial throat [47]. However, its excellent performances have not been applied on FET-biosensing yet.

Therefore, LIG, which is low-cost, process-simple, and suitable for large-scale fabrication, appears to be a promising alternative to fabricate graphene-based devices. The good electrical properties and synchronous manufacturing and patterning capability of LIG are ideal for the channel material of the FET. Meanwhile, the porous LIG with oyster-reef-like morphology can enlarge the binding area of biological molecules and reduce the influence of solvent flushing. The target protein can be directly absorbed on the surface of the LIG channel via graphene–protein affinity interaction [48], which benefits the electrical response of the biosensor when detecting the virus. In addition, PI, a flexible substrate, can be bent into different shapes and is suitable for wearable applications.

Herein, we have realized a one-step and low-cost LIG-FET biosensor. The structure of the LIG-FET is based on the liquid ion gate FET, of which the gate is also known as the reference electrode. The dielectric layer capacitor of the ion gate FET is composed of the reference electrode, the electrolyte solution, and the LIG channel. The ion gate FET modulates the carrier concentration in the channel through the gate voltage applied to the reference electrode. When the LIG-FET is functionalized with the SARS-CoV-2 spike antibody, it shows high sensitivity in detecting the SARS-CoV-2 virus. In this biosensing platform, the SARS-CoV-2 spike antibody was immobilized onto the device through 1×PBS (phosphate-buffer saline). Compared with the past graphene FETs [39], the sensitivity of the COVID-19 LIG-FET biosensor to the SARS-CoV-2 spike protein reaches a limit of detection (LOD) of 1 pg/mL. Furthermore, the LIG-FET could specifically distinguish the SARS-CoV-2 spike protein from the nucleocapsid protein in human serum. These results demonstrate a successful fabrication of a COVID-19 LIG-FET sensor, which has promising applications of fast, cheap, and highly sensitive detection of the SARS-CoV-2 virus.

## 2. Results and Discussion

### 2.1. Device Fabrication and Material Characterization

Laser-inducing technology realizes the fast-growth of porous graphene. A low-cost and portable 450 nm laser platform was chosen in this work for the laser-inducing. Figure 1b shows the one-step laser-inducing process to obtain the LIG. A PI film with a thickness of 200 μm is evenly laid under the laser platform, and the laser directly irradiates the PI film to convert it into LIG. A software platform decides the movement of the laser through the control unit so that a well-designed pattern can be transformed at the exact location on the PI substrate by laser irradiation. By adjusting the laser intensity and the scribing speed, the LIG with different structures and reduction degrees can be obtained.

The channel region of the LIG-FET was fabricated using an 840 mW laser, and the source/drain electrode region was fabricated using a 900 mW laser. After the laser-inducing process, the carbonization is clearly shown on the PI substrate. As shown in the photograph and the optical micrograph of the LIG-FET (Figure 1c,d), the LIG generated under a different laser power shows different colors due to different structures and reduction degrees of the LIG. The channel and source/drain regions show a porous microstructure with different expansion degrees. For the convenience of the electrical test, the silver paste was used to extract electrodes in the source/drain regions. After that, both electrodes were passivated with the SU-8 photoresist for protection. For the vast detection needs of the COVID-19 virus, the LIG-FET has a technological advantage in rapid mass production, as shown in Figure 1c. A LIG-FET array can be fabricated in minutes under a standard 450 nm UV-laser, which means each LIG-FET can be fabricated by a laser platform in seconds. The wiring and packaging methods conform to current techniques in the microelectronic industry, suitable for industrial production.

For the detection of the spike protein, the s-protein (spike protein) antibody is immobilized as the detection probe. After 15 min of combination [49], the s-protein antibody is fully bound to the LIG. Then, we rinse the residual s-protein antibody (which is not combined with graphene) with phosphate-buffer saline and use bovine serum albumin (BSA) as the blocking protein to block the unbound bonding points. The solvent of the s-protein antibody and BSA are all 1×PBS (pH = 7.9).

**Figure 2:**
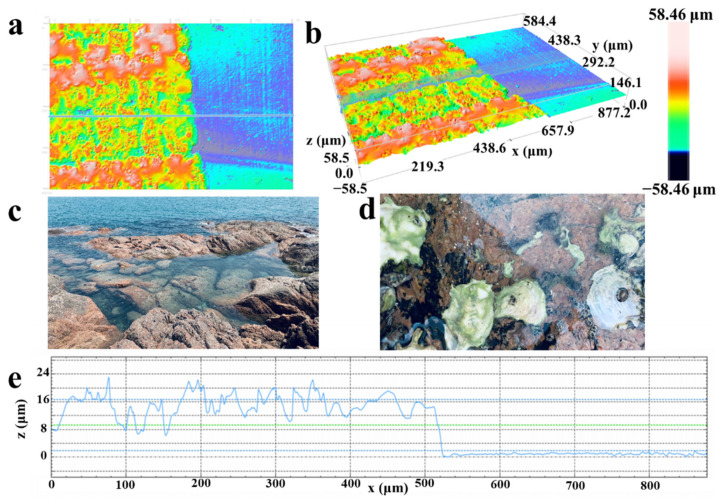
Morphology of the channel region in the LIG-FET. Images show 2D (**a**) and 3D (**b**) of the channel region by confocal microscopy, the left side is the LIG region, the right side is the PI substrate. (**c**) A photo of the oyster reefs in the costal, which looks like the morphology of the channel region in the LIG-FET, the state of the LIG channel after adding liquid gate dielectric layer is displayed macroscopically. (**d**) A photo of oysters binding to the reef. Macroscopically, the binding state of antibodies and LIG channel is shown. (**e**) The height measurement result of the transverse section (The blue line in Figure 2a,b) of the channel region, which shows the rough surface morphology of the LIG channel.

To study the morphology and composition of the PI films before and after laser irradiation, and the differences between LIG in channel region and drain/source region, the characterization of LIG was investigated with confocal microscopy, SEM, and Raman spectroscopy. From confocal microscopy images (Figure 2a,b), the LIG channel region looks quite like the reef in the sea (Figure 2c), with sponge-like porous morphology, which comes from the high-temperature heating during the laser irradiation process. During this process, the high temperature pyrolyzes the PI surface, breaks the C-O, C=O, and N-C bonds, and vents carbonaceous and nitric gases. The pyrolysis process continues until the temperature falls, and the pyrolysis time decides the depth of the porous structure. The resulting structure resembles reefs in the sea, which can provide a stable habitat for marine organisms which benefit from its porous surface. When facing a surge, oysters can bind tightly with the reefs (Figure 2d), and the porous surface helps prevent them from being flushed away [50,51]. Besides, the oysters filter large amounts of particulate matter from the water column. For the LIG-FET, the antibody probe is just like oysters which integrate tightly with the LIG ‘reef-like surface’. Moreover, the hybridization between s-protein antibodies and antigens has similarities with the denitrification process on oysters. In Figure 2a,b, the porous and rough structure of LIG is clearly shown in the three-dimensional image, which greatly enlarged the bounding area between the LIG and s-protein antibody, and prevented the desorption of biomolecules when the liquid gate dielectric layer is applied or removed. To present the profile of the LIG channel region, we obtained it by the cross-section in the blue line in Figure 2a,b. The obtained profile in Figure 2e shows the porous surface structure of the channel region; the left coarse surface is the LIG in the channel area, while the right flat surface is the PI substrate.

**Figure 3:**
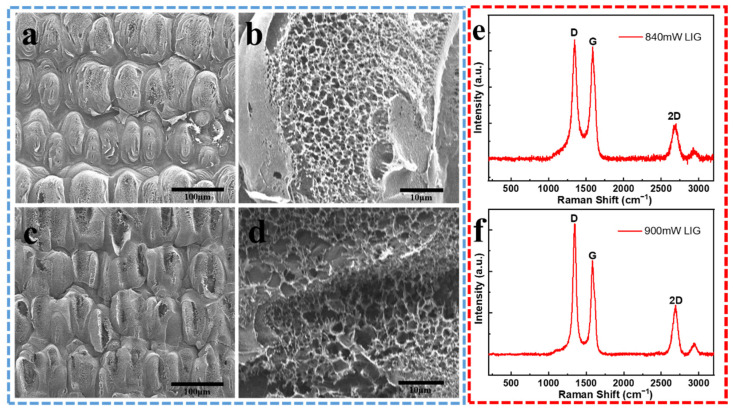
SEM images and Raman spectrum of the LIG-FET. (**a**) Top view of the LIG channel region. Scale bar, 100 μm. (**b**) Magnified image of the channel region with the small aperture porous structure of the 840 mW LIG, scale bar: 10 μm. (**c**) Top view of the source/drain region. Scale bar, 100 μm. (**d**) Magnified image of the source/drain region showing the large aperture porous structure of the 900 mW LIG. scale bar, 10 μm. (**e**) Raman spectrum of the LIG channel induced by 840 mW laser, and (**f**) the LIG electrode induced by 900 mW laser.

To further understand the formation of the LIG-FET, the morphology differences of the LIG in the channel region and source/drain region are shown by SEM images (Figure 3a–d). With different laser power, the channel region and source/drain region show morphological differences. Although both regions show porous fish-scale structure, the 900 mW laser-induced region (the channel region) shows a more porous and fluffy structure. From the high-magnification images (Figure 3b,d), the differences between the structures become more distinctive. Thin layers of carbonized films with reef-like morphology induced by the 900 mW laser are more porous and irregular than the films induced by the 840 mW laser, because of the higher temperature caused by the 900 mW laser during the laser irradiation process, which breaks more bonds of the PI substrate and release more carbonaceous and nitrogen gases [52]. The porous and fish-scale-like morphology in the channel region greatly enlarges the binding area of the s-protein antibody/antigen. Moreover, the high specific surface area of LIG benefits the electric connection between silver paste and source/drain electrodes.

The Raman spectrums (Figure 3e,f) of the LIG in channel and source/drain regions are performed to further investigate the laser irradiation effects and the composition change of the LIG. The spectrums obtained at both regions have similar characteristics, with a D-, G-, and 2D-peak at 1350, 1582, and 2700 cm^−1^ respectively. Since the LIG was made from laser-inducing, it has a porous, multilayer stacked graphene structure. The D-peak has a higher uplift at ~1350 cm^−1^, which is induced by defects or bent sp^2^-carbon bonds. The G-peak corresponds to the lattice vibration mode, and the 2D-peak at ~2700 cm^−1^ stems from second-order zone–boundary phonons. Compared with the Raman spectrum of random amorphous carbon [44], the spectrum of the LIG is different, which proves the existence of random graphene stacks in the LIG. When the laser intensity increases from 840 mW to 900 mW, the D/G intensity ratio increases, which indicates a high degree of graphene formation in the LIG films [53]. Besides, the increased G/2D intensity indicates more stacked graphene layers were created by the higher laser power. These results show the source/drain region has a higher degree of porosity and conductivity, suitable for electric connection.

### 2.2. Concentration Gradient Detection

Silver paste has been used as a source/drain extraction electrode to facilitate electrical measurements. The source/drain electrodes have been packaged by SU-8 for protection, which also limits the range of the liquid gate dielectric and the position of the Ag/AgCl gate electrode. After 15 min of incubation in the channel region at a time, the s-protein antibody forms the detection probe at first, and then the BSA blocks the remaining binding sites. Finally, by putting the Ag/AgCl reference electrode on the channel region, the LIG-FET can be used for biosensing.

Because of the bipolar character of graphene, electrons and holes are interchangeable. The gate voltage corresponding to the lowest point of the LIG-FET transfer characteristic curve is called the Dirac point, where the electron concentration equals the hole concentration. With the gate voltage increasing, the hole concentration decreases, and the electron concentration increases. During the detection, with the solution which contains the target molecules in contact with the LIG channel, the specific receptors modified on the LIG surface bind to the target molecules in 15 min [49]. Since the target generally carries an electric charge in the electrolyte solution, the charge carrier concentration in the LIG will change under the electrostatic field, causing the shift of the transfer characteristic curve. When the target molecules have a positive charge, the electron concentration in the LIG increases and the holes are depleted due to the electric field induction. This will lead to a negative shift in the transfer curve, which is shown by n-type doping of the LIG. At this point, if the gate voltage is fixed to a value less than the Dirac voltage, the left shift of the curve will lead to the decline of the channel current I_DS_, otherwise fixing the gate voltage to a value greater than the Dirac voltage will increase the I_DS_. With the increase in solution concentration, more charged targets will bind to the receptors, and the degree of curve bias will gradually increase. If the appropriate gate voltage is selected, the I_DS_ will monotonously change with the concentration of target molecules. Through this principle, the concentration of the targets in the solution can be obtained by measuring the current of the LIG-FET.

**Figure 4:**
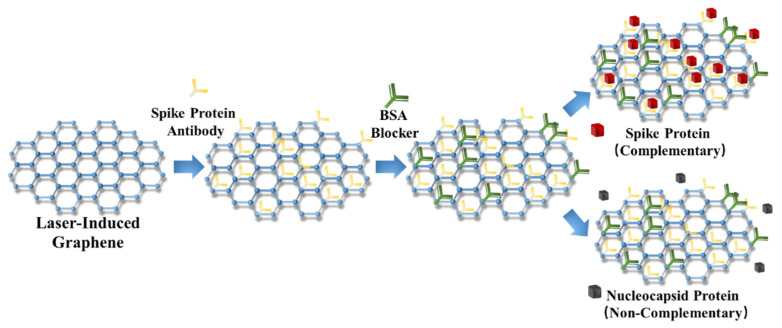
Illustration of the specificity detection by the LIG-FET based biosensor. After binding the spike protein antibody to LIG, and using BSA blocker to block other binding sites, the LIG-FET can realize specificity detecting of the spike protein and does not bind to non-complementary.

Figure 4 illustrates the working principle of the LIG-FET-based biosensor for COVID-19 virus s-protein detection. With the help of the 1 × PBS medium, the s-protein antibodies are combined with the LIG channel as the detection probes through π-π stacking interaction. After 15 min of incubation, the channel region is adequately flushed with the PBS solution and pumped dry to remove the residual and weakly bound antibodies. Then, the BSA blockers are loaded for blocking the binding sites which were not occupied by the s-protein antibody probes. After adequately flushing with PBS and pumping dry to remove the redundant BSA, the LIG-FET can be used for concentration gradient detection. As a detection target, 2 μL of s-protein solution with exact concentration is loaded on the channel region and captured by the s-protein antibody probes in 15 min. The hybridization of the complementary s-protein antibody and antigen causes n-doping of the devices because of the π-π stacking interaction between graphene and protein [54], which can be shown by the transfer characteristic curves of the FET before and after the incubation process between the LIG and the s-protein antibody/BSA. During the concentration gradient detection, once the s-proteins are captured by the probes, the Dirac point of the LIG-FET shifts, which corresponds to the concentration of the s-protein. Otherwise, if using the noncomplementary antigens, the hybridization process will not induce an apparent Dirac point shift. As a result, the s-protein detection realized by the LIG-FET is performed by monitoring the Dirac point shift in transfer characteristics.

**Figure 5:**
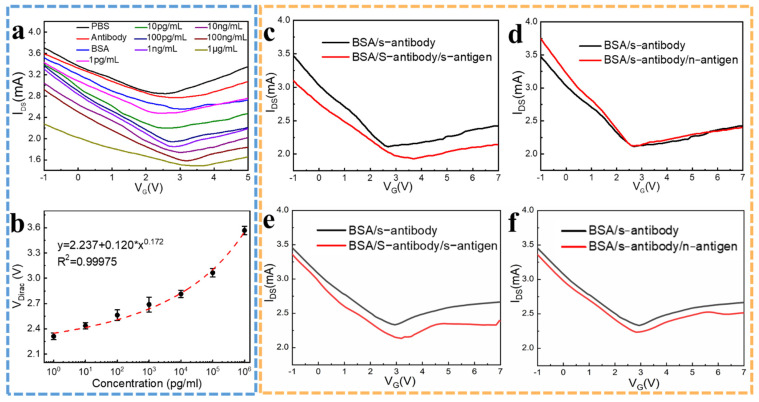
The virus detecting performance of the LIG-FET. (**a**) Transfer characteristics of the LIG-FET biosensor responding to gate solutions with different molecules and different concentrations of target spike protein. (**b**) V_Dirac_ dependence of the concentration of target spike protein corresponding to the LIG-FET biosensor. (**c**) Transfer characteristics of the LIG-FET biosensor responding to complementary 1 pg/mL spike protein in PBS solution, and (**d**) responding to noncomplementary 1 pg/mL nucleocapsid protein in PBS solution. (**e**) Transfer characteristics of the LIG-FET biosensor responding to 1 pg/mL complementary spike protein in human serum, and (**f**) responding to 1 pg/mL noncomplementary nucleocapsid protein in human serum.

A Keithley 2636 B digital multimeter was used for monitoring the transfer characteristic curve (I_DS_-V_G_). During the detection, a constant source-drain voltage of V_DS_ = 2 V was applied, and the gate voltage was swept from −5 V to +10 V while measuring the drain-source current I_DS_ through the LIG channel. The PBS solution was used as the solvent gate dielectric layer. After the integration of the LIG-FET and the SU-8 package, the quantitative samples in the PBS solution were added quantitatively with a pipetting gun onto the channel and were incubated at room temperature for 15 min before the test. After the incubation, the unbound/weak-bound protein molecules were flushed out in the channel area with PBS solution gently. After that, 2 μL PBS solution was dropped as the dielectric layer. Then, the gate electrode was put into the PBS solution to apply gate voltage. To verify the concentration detecting ability of the LIG-FET, spike protein solutions of 1 pg/mL, 10 pg/mL, 100 pg/mL, 1 ng/mL, 10 ng/mL, 100 ng/mL, and 1 μg/mL, were taken as samples, and the same amount of PBS solution was used as the blank control group. The transfer characteristic curve (I_DS_-V_G_) of the LIG-FET is shown in Figure 5a. At first, after the s-protein antibody incubation, the Dirac point has a 0.377 V right shift, which resulted from the n-type doping effect caused by the graphene–protein incubation. The BSA incubation process shows a similar n-type doping effect, with a 0.303 V right shift. When the s-protein hybridizes with the probe antibody, the V_Dirac_ of the device shifts upwardly with the increasing s-protein concentration, along with the channel resistance increasing. Meanwhile, the slope of the transfer curves decreases, indicating the decrease in both electron and hole mobility because of the increase in charge impurity scattering. The successful hybridization of antibodies and antigens results in more proteins being adsorbed on the graphene surface, which will result in the decrease in channel conductance (Figure 5a). The right shift of V_Dirac_ after s-protein antigen hybridization suggests that the complementary antigens can effectively interact with LIG and impose the n-doping effect based on the graphene-protein interaction. The detection sensitivity for s-protein determines the capability of the LIG-FET. Figure 5b shows the LIG-FET provides a reliable electrical signal for the detection of the target analytes. The sensitivity of the LIG-FET reaches 0.2 V/decade in the testing range with a correlation coefficient R^2^ = 0.99975. The relationship between the concentration x and the V_Dirac_ point conforms to the function V_Dirac_ = 2.237 + 0.120 × x^0.172^, which means the LIG-FET has a good quantization ability.

In this work, (i) s-protein hybridizes with specific antibody probes (s-protein antibody), and BSA is used to prevent the s-protein from nonspecific adsorption by LIG. The unbound or weak-bounded protein is flushed out by the PBS solution and pumped dry. These processes improve the detection limit, in which the s-protein antigen is hybridized with the s-protein completely. (ii) The s-protein and antibody can hybridize at room temperature in just 15 min, which reduces the desorption rate of the s-protein during hybridization and detection. (iii) LIG was used to improve the bonding area of the antibody probe and the s-protein antigen, which contribute to the large detection range, fast device fabrication, and inexpensive detection of COVID-19 virus.

### 2.3. Specificity Detection in PBS and Human Serum

Specificity sensing of the LIG-FET biosensor is crucial during practical applications, such as complex humoral or environmental samples, which contain not only the target protein but many interfering components. In this section, we first verified the specificity of the LIG-FET biosensor by using 1 pg/mL spike protein and nucleocapsid protein samples in PBS. Then by using 1 pg/mL spike protein and nucleocapsid protein samples in human serum, we further verified the specificity of the device in potential application environments. Finally, we compared the testing results.

To identify the specificity of the sensor, the s-protein was chosen as the target protein and the nucleocapsid protein (n-protein) was chosen as the noncomplementary target (Figure 4). The transfer characteristics of LIG-FET after the immobilization of the probes, and after hybridization with 1 pg/mL noncomplementary n-antigen and 1 pg/mL complementary s-antigen, were performed respectively. As shown in Figure 5c,d, when hybridized with 1 pg/mL complementary s-protein in PBS, the transfer curve (red line) shows an obvious right shift (Figure 5c), but when hybridized with 1 pg/mL noncomplementary n-protein in PBS, the transfer curve (red line) shows no significant change (Figure 5d). The specificity test results indicate that the LIG-FET-based biosensor is highly specific for the target COVID-19 virus spike protein. For future clinical use, considering the complex composition of the samples, we will further test the specificity of the LIG-FET using the 1 pg/mL spike protein and the nucleocapsid protein in real human serum, which also shows high specificity (Figure 5e,f). The LIG-FET-based biosensor is highly specific for the target COVID-19 virus protein in human serum, the transfer curve shows a significant shift with complementary s-protein and no significant change with noncomplementary n-protein. Although there are differences between the transfer curves of samples in the PBS solution and in human serum, which mainly attribute to the resistive difference between PBS solution and human serum, the V_Dirac_ points shift can indicate the specificity detection ability of LIG-FET. The results of the specificity tests indicate that the LIG-FET-based biosensor is highly specific for the target COVID-19 virus spike protein, leading to its clinical virus detecting applications.

## 3. Materials and Methods

The surface morphology of the LIG-FET was observed by a Quanta FEG 450 SEM (FEI Inc., Waltham, MA, USA). The confocal microscope was obtained by Leica confocal microscopy system (DCM8, Leica, Solms, Germany). Raman spectroscopy was performed using a laser with a wavelength of 532 nm (Renishaw inVia Raman Microscope, London, United Kingdom). The electrical signals of the LSG-FET were recorded by a digital source meter (Keithley 2636B, Cleveland, OH, USA). The gate of the biosensor is a single junction silver/silver chloride (Ag/AgCl) reference electrode which is filled with saturated KCl solution (Type-218, Leici, Shanghai, China), the gate electrode contacts with the liquid dielectric layer.

7.9 pH PBS (phosphate-buffered saline) was purchased from Corning (Corning, NY, USA) and used without further treatment. Recombinant COVID-19 spike-RBD protein (4.2 mg/mL), recombinant COVID-19 nucleocapsid protein (4.5 mg/mL), anti-COVID-19 spike Mab (2 mg/mL), human COVID-19 spike-RBD protein (1 pg/mL), and human COVID-19 nucleocapsid protein (1 pg/mL) were purchased from Smart-Lifesciences (Changzhou, China). The bovine serum albumin (BSA) was purchased from Sigma-Aldrich (St. Louis, MO, USA) and diluted by the PBS to 0.1 wt%. Laser-scribing graphene was grown using a 450 nm DIAOTU UV-laser (Shanghai, China) and the power intensity in the channel region is 840 mW, and 900 mW in the electrode region. The Kapton PI film with a thickness of 200 μm was purchased from DuPont (Wilmington, NC, USA). SU8-2150 photoresist was purchased from RDMICRO (Suzhou, China). The silver paste was purchased from TENON (Beijing, China). All chemicals in this work were analytical grade or highest purity available for direct use without further purification.

During the concentration gradient detection and the specificity detection in PBS and human serum, 2 μL of PBS solution is used as a liquid dielectric layer for testing. The Ag/AgCl reference electrode was placed on the top of the channel and used to measure the transfer characteristics of the biosensor, and the packaging made from SU8-2050 photoresist was used to limit the position of the gate and the dielectric layer. During the concentration gradient detection, the device was tested from low to high concentrations, all the sample solutions were diluted by PBS to different concentrations and homogenized with the help of a pipette and the Kylin–Bell centrifugal machine (Haimen, China). At each concentration test interval, the channel region was cleaned with adequate PBS and pumped to dry.

## 4. Conclusions

During the COVID-19 pandemic, the development of a highly sensitive and rapid bio-sensing device has become increasingly important. Based on LIG, we developed a COVID-19 LIG-FET sensor, in which the SARS-CoV-2 spike protein antibody conjugated to the laser-induced graphene channel as the sensing area. Based on its oyster reef-like LIG sensing area, the sensor was able to detect SARS-CoV-2 spike protein in PBS solvent and real serum of people with high sensitivity, up to 0.2 V/dec and with an LOD of 1 pg/mL, and the Dirac point shift shows a high degree of correlation to the spike protein concentration. Furthermore, the device exhibited no measurable cross-reactivity with a nucleocapsid protein antigen. Therefore, our functionalized LIG-based sensor platform provides simple, cheap, fast, and highly responsive detection of the SARS-CoV-2 virus spike protein. Our results have paved the way for novel COVID-19 testing, which helps early detection and treatment of the disease, and would be beneficial to control the current pandemic.

## Data Availability

Not applicable.

## Sample Availability

Samples of the compounds are not available from the authors.
